# Model reduction of strong-weak neurons

**DOI:** 10.3389/fncom.2014.00164

**Published:** 2014-12-16

**Authors:** Bosen Du, Danny Sorensen, Steven J. Cox

**Affiliations:** Department of Computational and Applied Mathematics, Rice UniversityHouston, TX, USA

**Keywords:** LGMD, predictor-corrector, quasi-active, proper orthogonal decomposition, discrete empirical interpolation

## Abstract

We consider neurons with large dendritic trees that are weakly excitable in the sense that back propagating action potentials are severly attenuated as they travel from the small, strongly excitable, spike initiation zone. In previous work we have shown that the computational size of weakly excitable cell models may be reduced by two or more orders of magnitude, and that the size of strongly excitable models may be reduced by at least one order of magnitude, without sacrificing the spatio–temporal nature of its inputs (in the sense we reproduce the cell's precise mapping of inputs to outputs). We combine the best of these two strategies via a predictor-corrector decomposition scheme and achieve a drastically reduced highly accurate model of a caricature of the neuron responsible for collision detection in the locust.

## 1. Introduction

Since Hodgkin and Huxley the neuroscience community has built mathematical models of cells, junctions and circuits as means to both synthesize existing knowledge and to drive further experiments. The complexity of both individual neurons and the networks in which they function has posed serious challenges to those in search of minimal models. The goal of neuronal model reduction is to arrive at a compact description of the cell's “function” and an efficient means of computing its response to physiological stimuli. This is typically accomplished by discovering a smaller equivalent dynamical system and discerning from this a smaller equivalent electrical circuit. See Brunel et al. (2014), Jadi et al. (2014) and Hedrick and Cox (2014) for recent surveys.

We continue our focus, on reduced single cell models that preserve the spatio-temporal structure of their inputs, by providing a detailed synthesis of the active reduction strategy of Kellems et al. (2010) with the quasi-active reduction strategy of Hedrick and Cox (2013). The synthesis is achieved via an elegant method of Rempe and Chopp (2006) for decoupling portions of complex cells and is applied to a caricature of the Lobula Giant Movement Detector (LGMD), the neuron, Peron et al. (2009), responsible for collision detection in the locust. The LGMD has a large, non-spiking dendritic tree that integrates visual input in a retinotopic fashion and funnels this signal to a well defined Spike Initiation Zone (SIZ). Although the structural morphology of the LGMD, and its inputs, has been carefully mapped it is not yet understood what distribution of active and passive conductances permits the cell to discern threatening from, seemingly similar, innocuous visual stimuli. It is hoped that a reduced model will constrain the large parameter space and accelerate the search through this space, and that it will lead to a compact description of the complex task of collision detection as implemented by the full LGMD. For a thorough investigation of the notion of *weak excitability* in the context of hippocampal pryamidal cells see Golding et al. (2001).

We build and test a detailed (879 compartments) model of the LGMD in §2.1, decouple its branches in §2.2, reduce its active branch in §2.3 and then its quasi-active branches in §2.4. We recouple these two small (3 dimensional) systems in §2.5 and in §3 demonstrate that the drastically reduced system retains the full integrative qualities of the original 879-dimensional model while running 20 times faster.

## 2. Materials and methods

**Figure 1:**
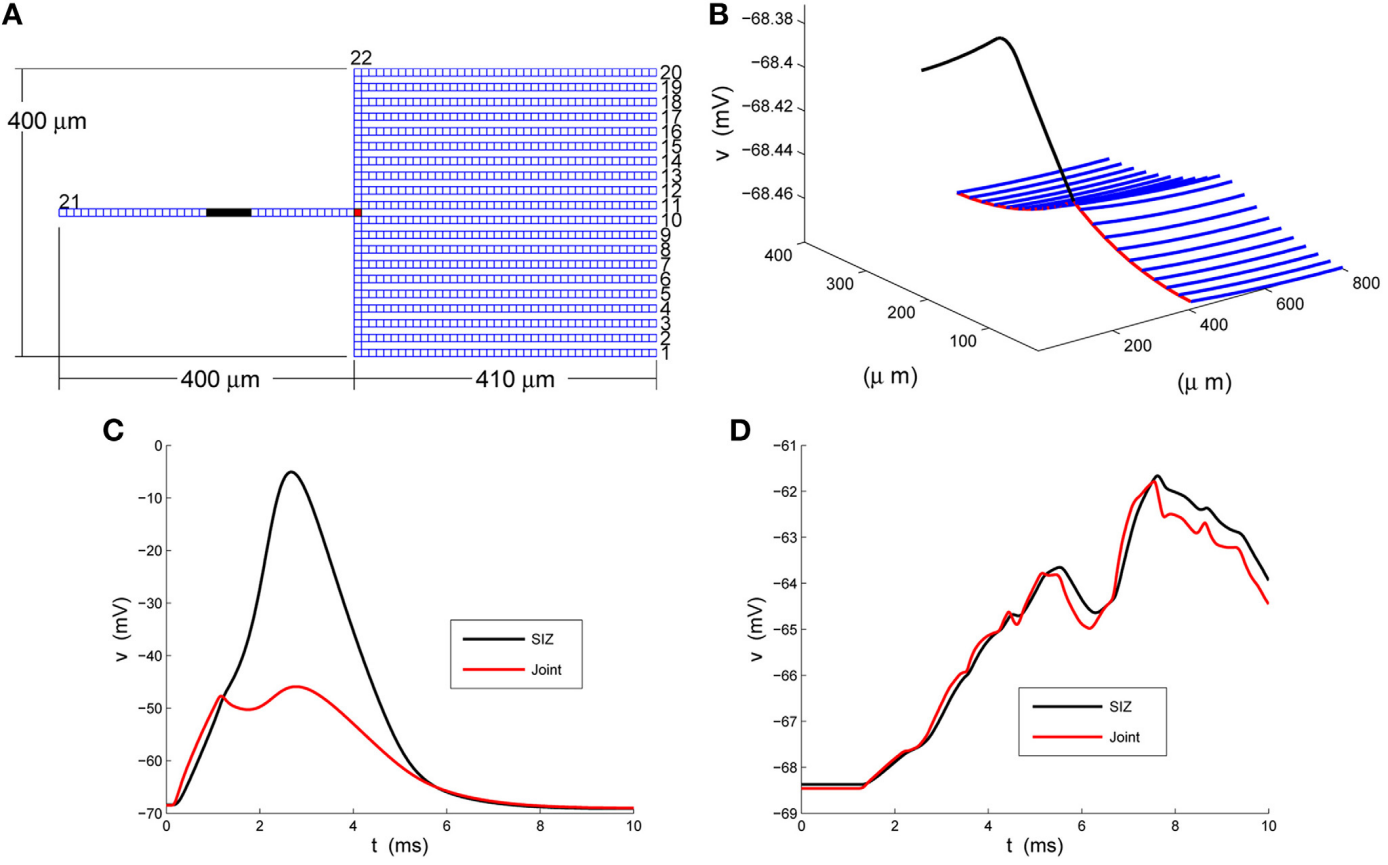
**A strong-weak neuron. (A)** The layout of the rake with its 22 numbered branches, divided into compartments of length *dx_j_* = 10 *μm*. **(B)** The rest potential, *v*, obtained by solving Equation 2 subject to the parameters specified in Equations 3 and 4. **(C)** The response of the full model, Equation 1, at the SIZ (left end of black region in **A**) and joint (red square in **A**) to the coherent synaptic input of Equation 5. **(D)** The response of the full model, Equation 1, at the SIZ (left end of black region in **A**) and joint (red square in **A**) to the random synaptic input of Equation 6.

The caricature of the LGMD neuron raised by Peron et al. (2009) is the rake depicted in Figure 1A. We have numbered its 22 branches and marked its SIZ, in black, near the center of the handle (branch 21) and the joint, in red, where the deck (branch 22) meets the handle. We have chosen a compartment (spatial step) size of *dx_j_* = 10 *μm* and so arrive at a base system with 879 compartments. These are illustrated in Figure 1A and their spatial dimensions are best seen in Figure 1B. We distribute standard sodium, potassium and chloride channels throughout the rake in such a fashion that the tines, branches 1 through 20, weakly integrate synaptic input, funnel it to the deck which then delivers it via the joint to a strongly excitable handle.

After specifying the full model we decompose it via a predictor-corrector scheme and then apply distinct reduction strategies to the strong and weak parts. Throughout we have used a time step of *dt* = 0.005 ms.

### 2.1. The full model

With regard to the rake depicted in Figure 1A, we suppose that the radius of the *j*th branch is *a_j_* = *a_j_*(*x*), where *x* denotes distance along the branch, and that its associated transmembrane potential is *v_j_* = *v_j_*(*x, t*). If the branch contains sodium, potassium and chloride ion channels and is subject to direct current stimulation then Kirchhoff's current law reads

(1) $$\begin{array}{l} C_{m}\partial_{t}v_{j}=\frac{1}{2a_{j}R_{a}}\partial_{x}(a_{j}^{2}\partial_{x}v_{j})+I_{stim,j}/(2\pi \;a_{j})\\ \;-\;g_{Na,j}m_{j}^{3}h_{j}(v_{j}-E_{Na})-g_{K,j}n_{j}^{4}(v_{j}-E_{K})\\ \;-\;g_{Cl,j}(v_{j}-E_{Cl})\\ \;\partial_{t}m_{j}\;=(m_{\infty }(v_{j})-m_{j})/\tau_{m}(v_{j}), \end{array}$$

where similar gating equations hold for *h_j_* and *n_j_*. In addition, we solve Equation 1 subject to sealed ends, current balance at branch points and initial conditions *v_j_*(*x*, 0) = *v_j_*(*x*) and *w_j_*(*x*, 0) = *w*_∞_(*v_j_*(*x*)) where *v_j_*(*x*) is the associated rest potential, obtained by solving

(2) $$\begin{array}{l} {\left(a_{j}^{2}(x){{\bar{v}}^{\prime }}_{j}(x)\right)}^{\prime }=2a_{j}R_{a}\{g_{Na,j}\;(x)m_{\infty }^{3}({\bar{v}}_{j}(x))h_{\infty }({\bar{v}}_{j}(x))({\bar{v}}_{j}(x)-E_{Na})\\ \;+\;g_{K,j}(x)n_{\infty }^{4}({\bar{v}}_{j}(x))({\bar{v}}_{j}(x)-E_{K})\\ \;+\;g_{Cl,j}(x)({\bar{v}}_{j}(x)-E_{Cl})\}, \end{array}$$

again subject to sealed ends and current balance at branch points. We concentrate throughout on a single set of parameters. The choice

(3) $$\begin{array}{l} C_{m}\;=1.5\mu F/cm^{2},\;R_{a}=0.05k\Omega cm,\;a_{j}=5\mu m\\ E_{Na}\;=56,\;E_{K}=-77,\;E_{Cl}=-68mV\\ g_{Na,j}=2\;g_{K,j}=3.6,\;g_{Cl,j}=0.9mS/cm^{2} \end{array}$$

will render the tines, branches 1–20, and the deck, branch 22, weakly excitable, while setting

(4) $$\begin{array}{l} g_{Na,21}(x)=\{\begin{array}{ll} 216\;mS/cm^{2}, & 200\leq x<260\mu m\\ 12\;mS/cm^{2}, & \text{otherwise}. \end{array}\\ \text{ and }g_{Cl,21}=0.3mS/cm^{2} \end{array}$$

will make the handle, branch 21, strongly excitable. We have illustrated the resulting rest potential, *v*, in Figure 1B. We see that the non-uniformity in Equation 4 leads to a depolarized handle and a non-uniform rest potential throughout the remainder of the rake.

We will solve this full system, Equation 1, for two classes of inputs. For the first class, deemed coherent, we simulataneously inject 4 nano-Amperes of current at the midpoint of each tine for nine tenths of a millisecond. In symbols

(5) $$I_{stim,j}(x,t)=0.004\delta (x-200)\;\chi_{\left[0.1,\;1\right]}(t),\;1\leq j\leq 20,$$

where χ_[*a, b*]_(*t*) equals one if *a* ≤ *t* ≤ *b* and equals zero otherwise. For the second class, deemed random, we inject 4 nano-Amperes of current at a random location, and at a random time, on each tine for nine tenths of a millisecond. In symbols

(6) $$I_{stim,j}(x,t)=0.004\delta (x-x_{j})\chi_{\left[t_{j},t_{j}+0.9\right]}(t),\;1\leq j\leq 20,$$

where the mean of *x_j_* is 200 *μm* and the mean of *t_j_* is 5 *ms*.

In response to coherent stimulus, Equation 5, we see in Figure 1C steady and significant (20 *mV*) depolarization at the joint (red trace) that is sufficient to drive the handle to spike (blue trace at SIZ). This spike travels down the handle and leads to the second, smaller, depolarization at the joint. The random stimulus, Equation 6, delivers the same amount of current to the rake but spread over space and time. The response at the joint, red trace in Figure 1D, indicate ≈3 *mV* depolarizations to individual current steps. These are not coherent enough to accumulate in a fashion sufficient to drive the handle to spike. Instead the response at the SIZ, blue trace in Figure 1D, is a filtered attenuated version of the joint trace.

### 2.2. Branch decomposition

Rempe and Chopp (2006) introduced a rational scheme for decomposing large cells into smaller (typically single branch) regions. They were motivated by the fact that as an action potential travels through a cell, branches on either side of the action potential are relatively quiet and so need not be simulated/computed. As such they devised branch-wise activity measures, in both Rempe and Chopp (2006) and Rempe et al. (2008), that allowed them to build a spatially adaptive numerical scheme that focused resources solely on active branches. One significant advantage of their decomposition is that it permits simultaneous/parallel updating of the active branches. This feature has been successfully exploited by Kozloski and Wagner (2011). Our use of Rempe and Chopp (2006) is however, quite different. For we use their scheme to partition the cell into strong and weak zones that may then be reduced by strategies specific to the dynamics consistent with such zones.

**Figure 2:**
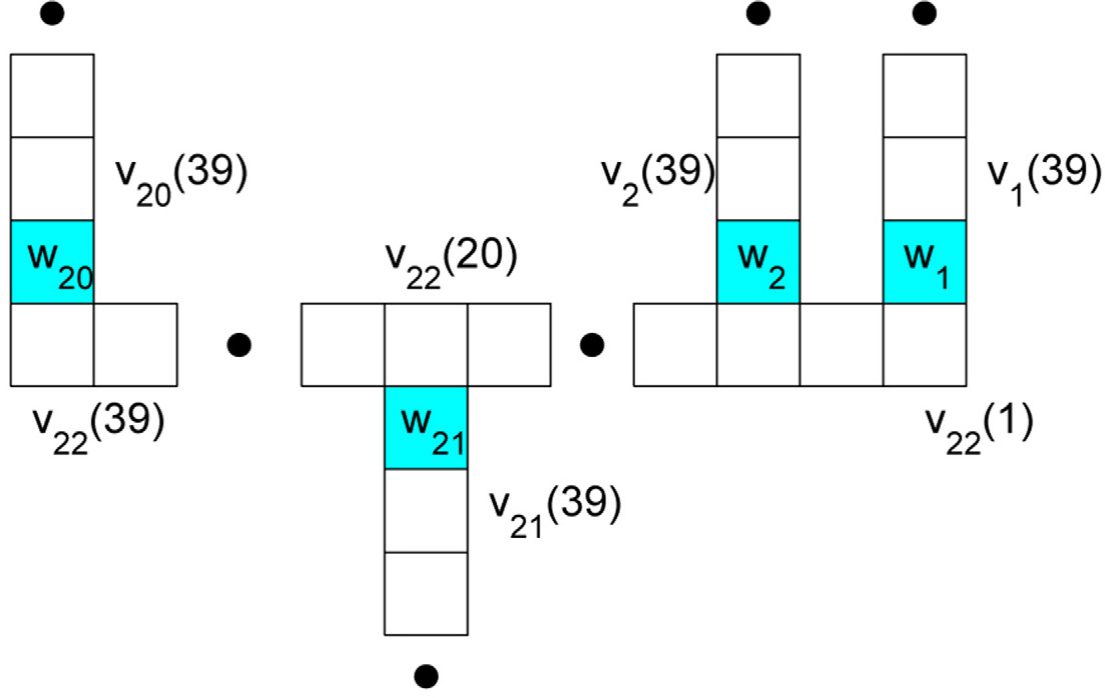
**Branch compartment and node labeling to facilitate decoupling via a predictor-corrector scheme**. The nodes are colored blue and their potentials are *w*_1_ through *w*_21_. They occur at the ends of 21 respective branches. The potential in compartment *k* of branch *j* is denoted *v_j_*(*k*).

Rempe and Chopp (2006) decompose the cell by giving special attention to those compartments, deemed **nodes**, at which branches meet. We have illustrated this decomposition on our rake in Figure 2. This spatial decomposition is only useful when coupled with a scheme for properly updating the components in time. Rempe and Chopp (2006) sketch a method that

1. uses the present branch and node potentials to **predict** the future node potentials,
2. updates the branch potentials based on the predicted node potentials,
3. **corrects** the node potentials based on the updated branch potentials.

As the success of our method hinges on this predictor-corrector scheme we present it here in some detail.

We distinguish between branches 1 through 21, which are adjacent to a single node, and branch 22, which is adjacent to many. Given the branch, *v_j_*(*k, t*), and node, *w_j_*(*t*), potentials and gating variables at time *t* we advance the gating variables via the explicit in *v* implicit in *m* step

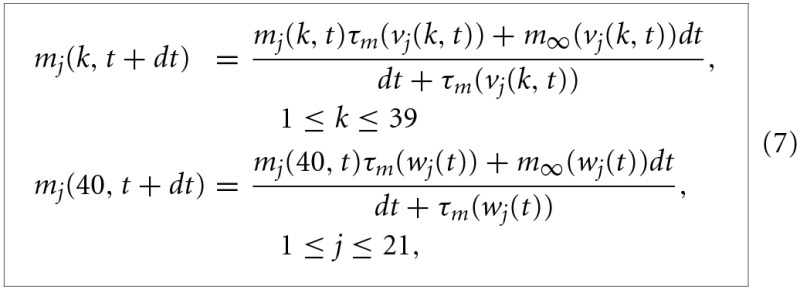



and collect these into

$$\begin{array}{l} \Gamma_{j}(k,t+dt)=g_{Na,j}(k)m_{j}^{3}(k,t+dt)h_{j}(k,t+dt)\\ \;+g_{K,j}(k)n_{j}^{4}(k,t+dt)+g_{Cl,j}(k)\\ \gamma_{j}(k,t+dt)=g_{Na,j}(k)m_{j}^{3}(k,t+dt)h_{j}(k,t+dt)E_{Na}\\ \;+g_{K,j}(k)n_{j}^{4}(k,t+dt)E_{K}+g_{Cl,j}(k)E_{Cl}. \end{array}$$

We next use these to take a backward Euler step of the associated voltage equation, Equation 1,

$$\begin{array}{l} G_{j}(k-)v_{j}(k-1,t+dt)-(G_{j}(k-)+G_{j}(k+))\\ v_{j}(k,t+dt)+G_{j}(k+)v_{j}(k+1,t+dt)=\\ \;\mu (v_{j}(k,t+dt)-v_{j}(k,t))+\Gamma_{j}(k,t+dt)v_{j}(k,t+dt)\\ \;-\gamma_{j}(k,t+dt)+I_{stim,j}(k,t+dt)/A_{j}(k)\; \end{array}$$

where

$$\begin{array}{l} G_{j}(k\pm )=\frac{a_{j}(k)}{R_{a}dx_{j}^{2}}\frac{a_{j}^{2}(k\pm 1)}{a_{j}^{2}(k)+a_{j}^{2}(k\pm 1)},\;\mu =C_{m}/dt\\ \text{ and }A_{j}(k)=2\pi a_{j}(k)dx_{j}. \end{array}$$

While at the ends, *G_j_*(1 −) = 0 and

$$\begin{array}{l} G_{j}(40-)\equiv G_{j-}^{*}=\frac{a_{j}(40)}{R_{a}dx_{j}^{2}}\frac{a_{j}^{2}(39)}{a_{j}^{2}(40)+a_{j}^{2}(39)}\\ G_{j}(40+)\equiv G_{j+}^{*}=\frac{a_{j}(40)}{2R_{a}dx_{j}}\frac{dx_{22}^{2}}{2a_{j}^{2}(40)dx_{j}+dx_{22}^{2}a_{22}(p_{j})}\\ \text{ where }p_{j}=\{\begin{array}{ll} 2j-1, & 1\leq j\leq 20\\ 20, & j=21. \end{array} \end{array}$$

With these we may now make sense of the node equation

(8) $$\begin{array}{l} G_{j-}^{*}v_{j}(39,t+dt)-(G_{j-}^{*}+G_{j+}^{*})\\ w_{j}(t+dt)+G_{j+}^{*}v_{22}(p_{j},t+dt)=\\ \;\mu (w_{j}(t+dt)-w_{j}(t))+\Gamma_{j}(40,t+dt)\\ \;w_{j}(t+dt)-\gamma_{j}(40,t+dt) \end{array}$$

Following Rempe and Chopp (2006) we decouple the node and branch equations in time by making a crude prediction of the nodal potentials by replacing the backward Euler step Equation 8 with the forward Euler step

(9) $$\begin{array}{l} G_{j-}^{*}v_{j}(39,t)-(G_{j-}^{*}+G_{j+}^{*})w_{j}(t)+G_{j+}^{*}v_{22}(p_{j},t)=\\ \;\mu (w_{j}^{*}(t)-w_{j}(t))+\Gamma_{j}(40,t+dt)w_{j}^{*}(t)-\gamma_{j}(40,t+dt) \end{array}$$

where w_j_^*^(*t*) denotes our crude prediction of *w_j_*(*t* + *dt*). We note that Equation 9 may be solved explicitly for

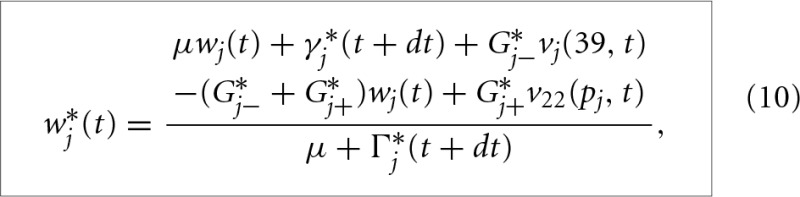



where Γ^*^_j_(*t* + *dt*) = Γ_*j*_(40, *t* + *dt*) and γ^*^_j_(*t* + *dt*) = γ_*j*_(40, *t* + *dt*). We then use these predicted nodal potentials to drive the branch updates via

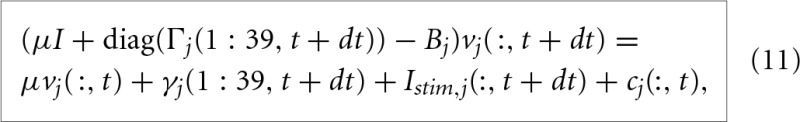



where *c_j_* is the node branch coupling vector and *B_j_* is the branch tridiagonal matrix. For the branches adjacent to a single node, *j* < 22, we find that *c_j_* is zero at each compartment except for

(12) $$c_{j}(39,t)=G_{j-}^{*}w_{j}^{*}(t),$$

while *B_j_* is the tridiagonal matrix

$$\begin{array}{l} \;B_{j}(1,1:2)=[-G_{j}(1+\text{​})\;G_{j}(1+\text{​})]\\ B_{j}(k,k-1:k+1)=[G_{j}(k-\text{​})\;-(G_{j}(k-\text{​})+G_{j}(k+\text{​}))\\ \;G_{j}(k+\text{​})],\;1<k<39,\\ \;B_{j}(39,38:39)=[G_{j}(39-\text{​})\;-(G_{j}(39-\text{​})\\ \;+G_{j}(39+))]. \end{array}$$

Turning to the deck, *B*_22_ is the tridiagonal matrix

$$\begin{array}{l} \;B_{22}(1,1:2)=[-(G_{22}(1+\text{​})+G_{1+}^{*}\text{​})\;G_{j}(1+\text{​})]\\ \;{\;}_{22}(k,k-1:k+1)=[G_{22}(k-\text{​})\;-(G_{22}(k-\text{​})\\ \;+G_{22}(k+\text{​}))\;G_{22}(k+\text{​})],\\ \;1<k<39,\;k\neq p_{j}\\ B_{22}(k,k-1:k+1)=[G_{22}(k-\text{​})\;-(G_{22}(k-\text{​})\\ \;+G_{22}(k+\text{​})+G_{j+}^{*})\;G_{22}(k+\text{​})],\\ \;1<k<39,\;k=p_{j}\\ \;B_{22}(39,38:39)=[G_{22}(39-\text{​​})\;-(G_{22}(39-\text{​})+G_{20+}^{*})]. \end{array}$$

This differs from the previous *B_j_* in the sense that it has no free ends (hence 2 terms on the end diagonals) and meets the 20 tines (and hence three terms on those diagonals). The associated coupling term is then zero except at

(13) $$c_{22}(p_{j},t)=G_{j+}^{*}w_{j}^{*}(t),\;j=1,\ldots ,21.$$

Upon updating all branches we may then return to correcting the nodal potentials, now via

(14) $$\begin{array}{l} G_{j-}^{*}v_{j}(39,t+dt)-(G_{j-}^{*}+G_{j+}^{*})\\ w_{j}(t+dt)+G_{j+}^{*}v_{p}(p_{j},t+dt)=\\ \;\mu (w_{j}(t+dt)-w_{j}^{*}(t))+\Gamma_{j}^{*}(t+dt)\\ \;w_{j}(t+dt)-\gamma_{j}^{*}(t+dt) \end{array}$$

which we solve explicitly for

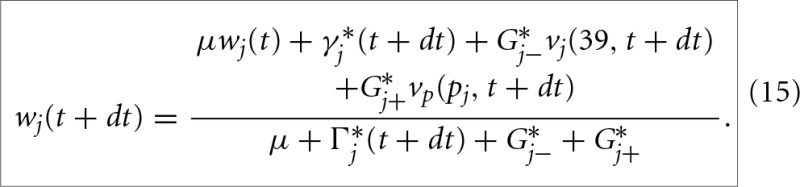



With this we may now offer a precise specification of the Predictor-Corrector Algorithm

1. Given the branch potentials, node potentials and gating variables at time *t* update the gating variables per Equation 7.
2. Predict the new values of the node potentials via Equation 10.
3. Update the branch potentials via Equation 11.
4. Correct the node potentials via Equation 15. Return to step [1].

### 2.3. Reduction of the strong part

Following Kellems et al. (2010) we reduce the dynamics in the strong zone, (*v*_21_, *m*_21_, *h*_21_, *n*_21_) by the method of Proper Orthogonal Decomposition by collecting snapshots of the membrane potential and associated active current

$$\begin{array}{l} I_{act}(t)\equiv g_{Na,21}.m_{21}^{3}(t).h_{21}(t).(v_{21}(t)-E_{Na})\\ \;+g_{K,21}.n_{21}^{4}(t).(v_{21}(t)-E_{K}) \end{array}$$

in

$$\begin{array}{l} V=[v_{21}(0)\;v_{21}(dt)\;\cdots \;v_{21}(T_{fin})]\text{ and }\\ F=[I_{act}(0)\;I_{act}(dt)\;\cdots \;I_{act}(T_{fin})] \end{array}$$

under a stimulus regime that generates a spike on branch 21. The major features of spike generation and propagation are purportedly captured in the first few singular vectors of *V* and *F*. Accordingly we compute the respective singular value decompositions

(16) $$V=U\Sigma A^{T}\text{ and }F=W\Lambda C^{T},$$

where the matrices of singular vectors, *U, A, W* and *C*, are orthonormal and the matrices of singular values, Σ and Λ, are diagonal – and ordered in a decreasing manner.

Our first stab at reduction is to suppose that *v*_21_ is well approximated by the first κ columns of *U*, i.e.,

$$v_{21}(t)\approx U_{\kappa }{\hat{v}}_{21}(t)$$

where *U*_κ_ denotes the first κ columns of *U* (from Equation 16) and so the reduced state $\hat{v}$_21_(*t*) ∈ ℝ^κ^. On placing this guess in the (spatially discretized version) of Equation 1 we find that the reduced state, $\hat{v}$_21_, must obey

(17) $$\begin{array}{l} C_{m}{{\hat{v}}^{\prime }}_{21}=U_{\kappa }^{T}B_{21}U_{\kappa }{\hat{v}}_{21}-U_{\kappa }^{T}\{g_{Na,21}.m_{21}^{3}.h_{21}.(U_{\kappa }{\hat{v}}_{21}-E_{Na})\\ \;+\;g_{K,21}.n_{21}^{4}.(U_{\kappa }{\hat{v}}_{21}-E_{K})+g_{Cl,21}(U_{\kappa }{\hat{v}}_{21}-E_{Cl})-c_{21}\}\\ {m^{\prime }}_{21}=(m_{\infty }(U_{\kappa }{\hat{v}}_{21})-m_{21})./\tau_{m}(U_{\kappa }{\hat{v}}_{21}). \end{array}$$

This provides a clean reduction of the linear spatial coupling between compartments, in the sense that

$${\tilde{B}}_{21}\equiv U_{\kappa }^{T}B_{21}U_{\kappa }$$

is merely κ-by-κ. The non-linearities however are still computed on the full dimensional vector *U*_κ_$\hat{v}$_21_. To address this we distil from *W*_κ_, the first κ columns of W (from Equation 16), κ places along the handle at which it suffices to evaluate the non-linear gating functionals. These places are selected by Discrete Empirical Interpolation as those places at which the singular vectors of *F* have the greatest content. In particular,

$$\begin{array}{l} z_{1}=\text{argmax}|W_{\kappa }(\text{​}:,1)|\\ P=e_{z_{1}}\\ \text{for }i=2:\kappa \\ \;s=(P^{T}W_{\kappa }(\text{​}:,1:i-1))\backslash P^{T}W_{\kappa }(\text{​}:,i)\\ \;r=W_{\kappa }(\text{​}:,i)-W_{\kappa }(\text{​}:,1:i-1)s\\ \;z_{i}=\text{argmax}|r|\\ \;P=[P\;e_{z_{i}}]\\ \text{ end} \end{array}$$

where *e_k_* denotes the *k*th column of the identity matrix on ℝ^39^. With these κ places, *z* = [*z*_1_, …, *z*_κ_] and their associated permutation matrix *P* we reduce the gating variables via

$$m_{21}(t)\approx P{\hat{m}}_{21}(t).\;h_{21}(t)\approx P{\hat{h}}_{21}(t)\text{ and }n_{21}(t)\approx P{\hat{n}}_{21}(t)$$

and so bring Equation 17 to

(18) $$\begin{array}{l} C_{m}{{\hat{v}}^{\prime }}_{21}={\tilde{B}}_{21}{\hat{v}}_{21}-R\{g_{Na}(z).{\hat{m}}_{21}^{3}.{\hat{h}}_{21}.(Z{\hat{v}}_{21}-E_{Na})\\ \;+\;g_{K}(z).{\hat{n}}_{21}^{4}.(Z{\hat{v}}_{21}-E_{K})\}-U_{\kappa }^{T}g_{Cl,21}(U_{\kappa }{\hat{v}}_{21}-E_{Cl})\\ \;+\;U_{\kappa }^{T}c_{21}\\ \;{{\hat{m}}^{\prime }}_{21}=(m_{\infty }(Z{\hat{v}}_{21})-{\hat{m}}_{21})./\tau_{m}(Z{\hat{v}}_{21}) \end{array}$$

where *g_Na_*(*z*) denotes the evaluation of *g*_*Na*, 21_ at the compartments indexed by *z*. As both

$$R=U_{\kappa }^{T}W_{\kappa }{\left(P^{T}W_{\kappa }\right)}^{-1}\text{ and }Z=P^{T}U_{\kappa }$$

are κ-by-κ we have arrived at a κ-dimesional reduction of the original 39-dimensional active handle. We solve Equation 18, subject to the initial conditions $\hat{v}$_21_(:, 0) = *U*^*T*^_κ_*v* and $\hat{m}$_21_(:, 0) = *m*_∞_(*Z*$\hat{v}$_21_(:, 0)), via the standard explicit-implicit Euler method

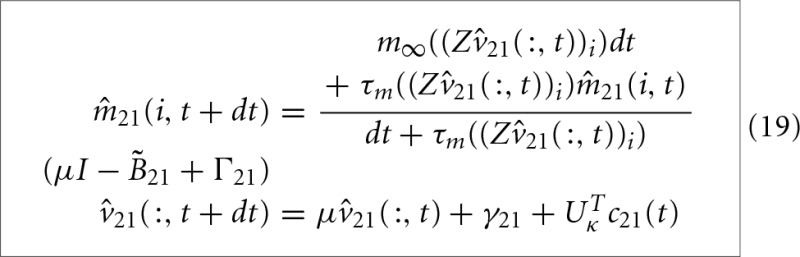



where

$$\begin{array}{l} \Gamma_{21}=R\text{diag}(g_{Na}(z).{\hat{m}}_{21}^{3}(\text{​}:,t+dt).{\hat{h}}_{21}(\text{​}:,t+dt)\\ \;+\;g_{K}(z).{\hat{n}}_{21}^{4}(\text{​}:,t+dt))Z+U_{\kappa }^{T}\text{diag}(g_{Cl,21})U_{\kappa }\\ \gamma_{21}=R(g_{Na}(z).{\hat{m}}_{21}^{3}(\text{​}:,t+dt).{\hat{h}}_{21}(\text{​}:,t+dt)E_{Na}\\ \;+\;g_{K}(z).{\hat{n}}_{21}^{4}(\text{​}:,t+dt)E_{K})+E_{Cl}U_{\kappa }^{T}g_{Cl,21} \end{array}$$

The *c*_21_ term in Equation 19 remains the contribution from the nodal potential, *w*_21_. Before specifying this we discuss how to reduce the remainder of the branches.

### 2.4. Reduction of the weak part

In order to perform a single reduction on the remaining branches it is most convenient to gather the variables in the 800 tine compartments and 39 deck compartments into four long vectors *v, m, h* and *n*. The first step is then to linearize the full system, Equation 1, about its rest state. More precisely, assuming the stimulus to be order ε we develop the voltage and gating variables

(20) $$\begin{array}{l} \;v(x,t)=\bar{v}(x)+\varepsilon \tilde{v}(x,t)+O(\varepsilon^{2})\\ \text{ and }m(x,t)=m_{\infty }(\bar{v}(x))+\varepsilon \tilde{m}(x,t)+O(\varepsilon^{2}). \end{array}$$

On substituting Equation 20 into Equation 1 and identifying terms of order ε, we find that the so-called quasi-active variables, $\tilde{v}$, $\tilde{m}$, $\tilde{h}$ and $\tilde{n}$ must solve

(21) $$\begin{array}{l} C_{m}\partial_{t}\tilde{v}=\frac{1}{2R_{a}a}\partial_{x}(a^{2}\partial_{x}\tilde{v})-(g_{Na,j}m_{\infty }^{3}(\bar{v})h_{\infty }(\bar{v})+g_{K,j}n_{\infty }^{4}(\bar{v})\\ \;+\;g_{Cl,j})\tilde{v}-3g_{Na,j}m_{\infty }^{2}(\bar{v})h_{\infty }(\bar{v})(\bar{v}-E_{Na})\tilde{m}\\ \;-\;g_{Na,j}m_{\infty }^{3}(\bar{v})(\bar{v}-E_{Na})\tilde{h}-4g_{K,j}n_{\infty }^{3}(\bar{v})(\bar{v}-E_{K})\tilde{n}\\ \;+\;I_{stim}/(2\pi a)\\ \;\partial_{t}\tilde{m}=({m^{\prime }}_{\infty }(\bar{v})-\tilde{m})/\tau_{m}(\bar{v}) \end{array}$$

subject to current balance where the tines meet the deck and to the initial conditions $\tilde{v}$(*x*, 0) = $\tilde{m}$(*x*, 0) = 0. On stacking the quasi-active variables in

$$y=[\tilde{m};\tilde{h};\tilde{n};\tilde{v}]$$

and the stimuli and coupling vector in

(22) $$u=[I_{stim,1};I_{stim,2};\cdots ;I_{stim,20};c_{22}]$$

we may write Equation 21 as an (839 · 4)–dimensional system for *y*,

(23) $$y^{\prime }(t)=Qy(t)+Bu(t),\;y(0)=0$$

where the non-zero blocks of *Q* and *B* are

$$Q=\left(\begin{array}{cccc} D_{m,1} & & & D_{m,2}\\ & D_{h,1} & & D_{h,2}\\ & & D_{n,1} & D_{n,2}\\ D_{v,1} & D_{v,2} & D_{v,3} & H \end{array}\right)\text{ and }B=\left(\begin{array}{c} I/C_{m} \end{array}\right).$$

See §9.4 of Gabbiani and Cox (2010) for the diagonal *D* matrices and the Hines matrix, *H*. Given the geometry of the rake we are really only interested in the potential at the joint (recall the red square in Figure 1A). As this is compartment number 820 in the natural ordering, out of the large system Equation 23 we ask only for a good approximation to the joint potential

$$J(t)=e_{839\cdot 3+820}^{T}y,$$

where *e_n_* is the unit vector with a one in element *n*. Following Hedrick and Cox (2013) we suppose *y* ≈ 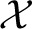
*ŷ* and choose an 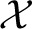, with only 4κ columns, that returns an accurate approximation of *J*. This is done by matching the first κ moments of the full and reduced transfer functions, or, equivalently, via the Arnoldi scheme

(24) $$\begin{array}{l} x=H\backslash e_{820}\;\\ X=x/\|x\|\;\;\;\\ \text{for }i=1:\kappa -1\\ \;x=H\backslash X(\text{​}:,i)\\ \text{ for }\;\;\;j=1:i\\ \;x=x-(X{\left(\text{​}:,j\right)}^{T}x)X(\text{​}:,j)\\ \text{ end}\;\;\;\;\;\\ \;X=[X\;x/\|x\|]\\ \text{end}\;\;\;\;\;\;\;\;\;\;\;\;\;\;\; \end{array}$$

where, for simplicity, we have chosen our reduced dimension, κ, to agree with that used to reduce the cell's strong zone. We then arrive at the full reducer by tiling this *X*, i.e.,

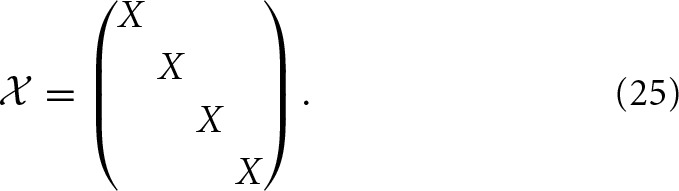



On inserting *y* = 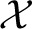
*ŷ* into Equation 23 and using 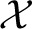
^*T*^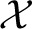 = I we find that *ŷ* must obeys

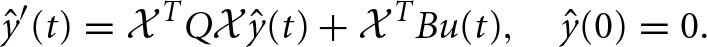



This we solve by backward Euler

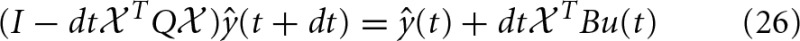



and then read off the approximate joint potential via $\hat{J}$(*t*) = *e*^*T*^_839·3+820_
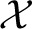
*ŷ*(*t*).

### 2.5. The reduced strong-weak neuron

It remains only to specify the predictor and corrector updates of the single nodal potential, *w*_21_, and to clarify their roles in the coupling vector *c*_21_ appearing in the strong reduction, Equation 19, and the coupling vector *c*_22_ in the weak reduction, Equation 26, via its presence in the *u* of Equation 22.

Regarding the expressions for w^*^_21_ and *w*_21_ in Equation 10 and 15 we note that the required adjacent potentials are readily derived from our independent reductions,

$$v_{21}(39,t)\approx U_{\kappa }(39,:)\hat{v}(t)\text{ and }v_{22}(20,t)\approx {\bar{v}}_{22}(20)+\hat{J}(t).$$

The coupling vector *c*_21_ is all zero except *c*_21_(39, *t*) = G^*^_21−_w^*^_21_(*t*), while the coupling vector *c*_22_ is all zero except *c*_22_(20, *t*) = G^*^_21+_(w^*^_21_(*t*) − *v*_22_(20)).

## 3. Results

**Figure 3:**
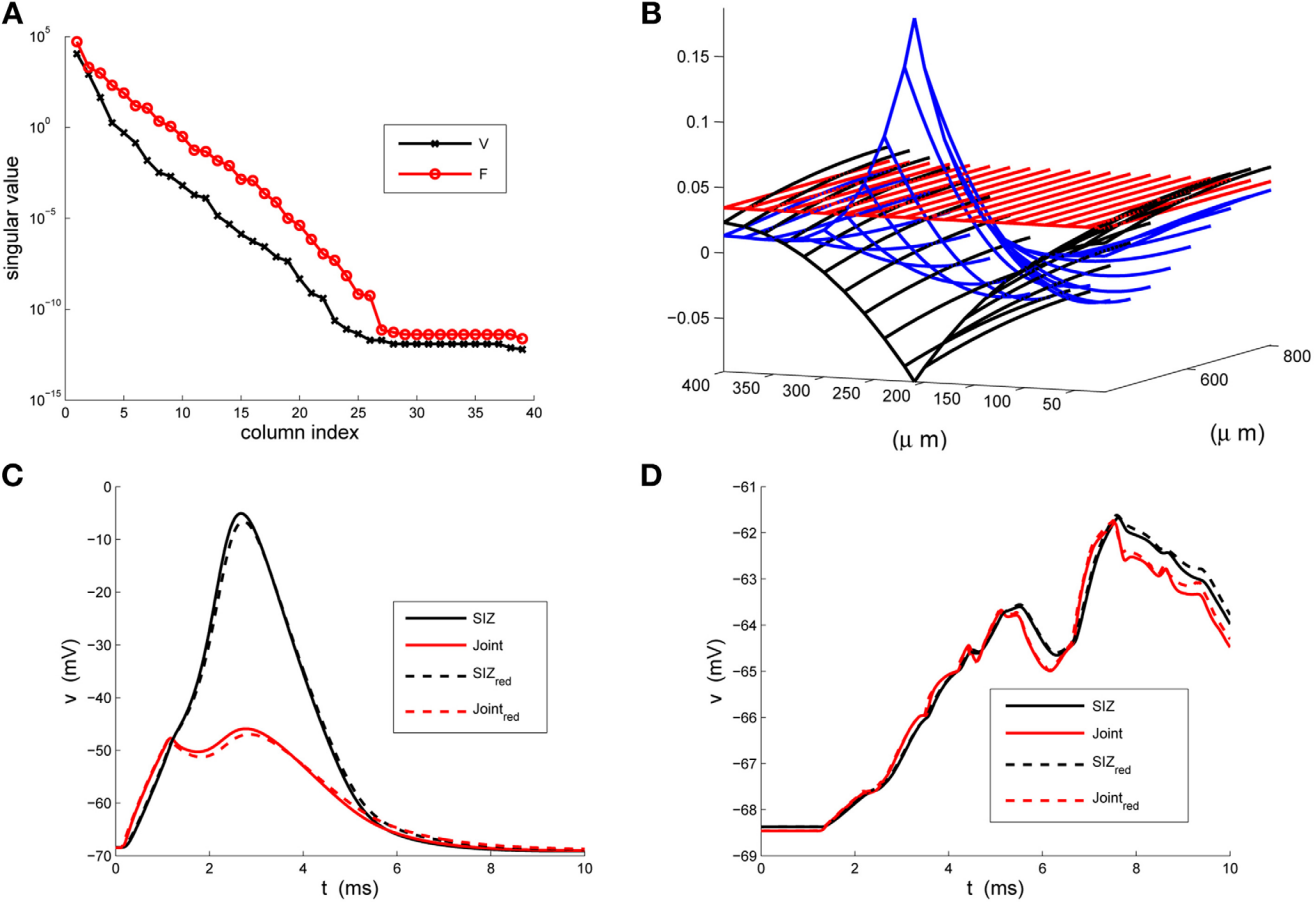
**The strong-weak reduction of the rake. (A)** The singular values of the voltage (V) and active current (F) snapshots. We see that both have decreased by two orders of magnitude by their third index. **(B)** An illustration of the three columns of the reducer, *X*, computed in Equation 24, indicating how the reduced system processes the true inputs. **(C)** Contrasting the response of the full (solid) and reduced (κ = 3) models at the SIZ (black) and at the Joint (red) to identical coherent stimuli. **(D)** Contrasting the response of the full (solid) and reduced (κ = 3) models at the SIZ (black) and at the Joint (red) to identical random stimuli.

We present in Figure 3 structural components of the strong (A) and weak (B) reductions and in panels (C) and (D) contrast the responses of the full and reduced models, at SIZ and Joint, to the respective coherent and random stimuli used in Figure 1.

**Figure 4:**
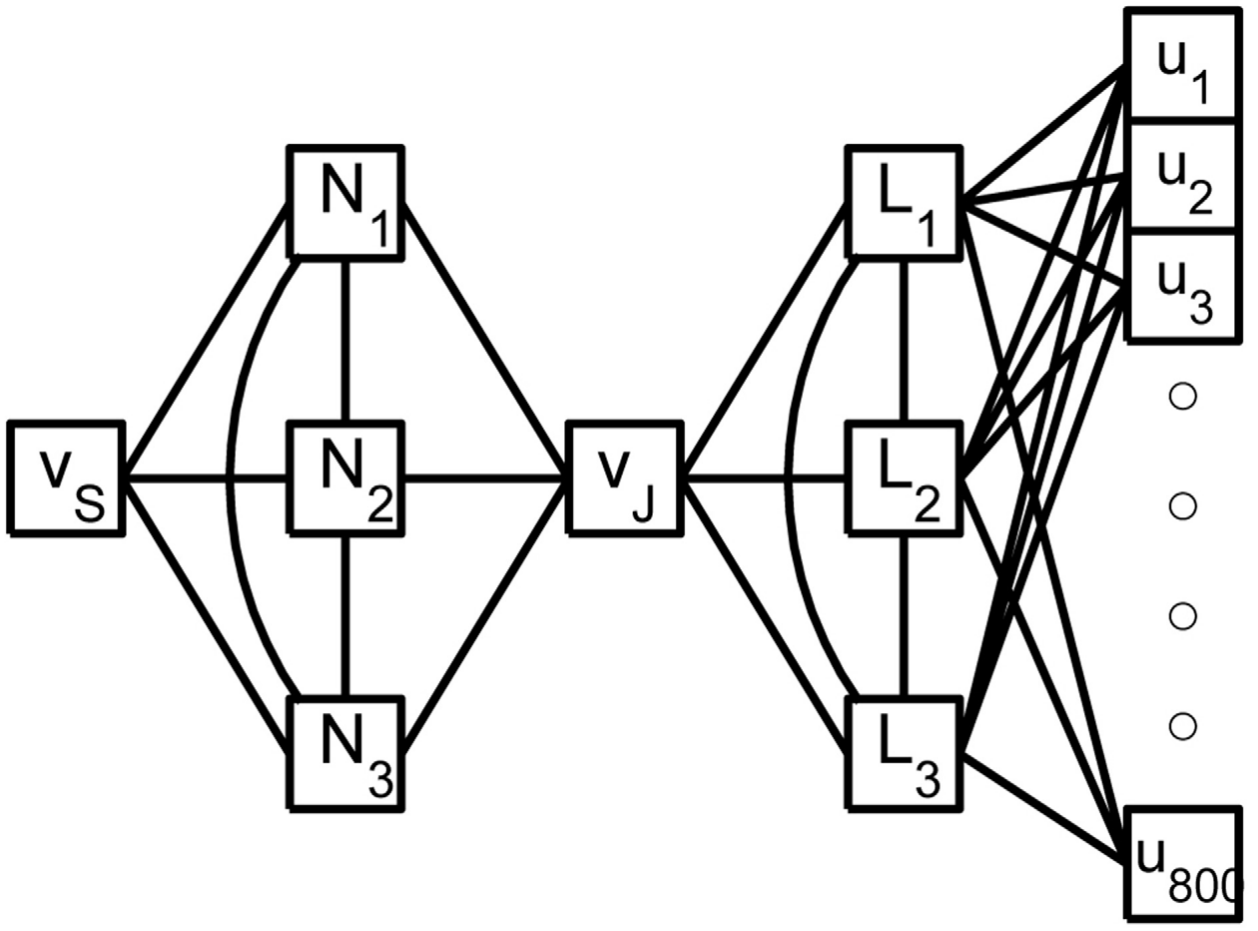
**A shematic of the reduced rake**. The true inputs, *u_i_*, into the 800 tine compartments are weighted by the columns of *X* and then summed as they enter the 3 linear nodes, *L_i_*, of the reduced weak zone. The linear nodes are fully coupled and a linear combination of their responses contributes to the joint potential, *v_J_*. The 3 fully coupled non-linear nodes, *N_i_*, of the reduced strong zone contribute to both *v_J_* and the SIZ potential *v_S_*.

These results were robust to changes in the stimuli that generated the snapshots and to changes in the random stimuli, Equation 6. The reduced system consistently ran in less than 1/20^*th*^ of the time required by the original system. With κ = 3 the Discrete Empirical Interpolation method identified *z* = [1, 21, 26] as the compartments along the handle at which to evaluate the gating variables. On comparison to the sodium channel distribution in Equation 4 we note that compartments 21 and 26 are the extent of the SIZ. Regarding Figure 3B we interpret the columns of *X* as the dendritic filter, seen at the joint, of the true inputs. For columns of *X* and biophysical interpretations of the elements of 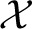
*^T^Q*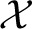 see Hedrick and Cox (2013). The errors reported in Figures 3C,D are quite small relative to the original signals in Figures 1C,D and, regarding the timing of critical SIZ events, produce negligible (< 0.1 ms) errors. This then permits us to replace the complex 879 compartment model of Figure 1A with the 8 compartment model of Figure 4.

## 4. Discussion

We have developed and demonstrated a strategy for the systematic reduction of models of strong-weak neurons. In particular, we have replaced a sensory neuron of dimension 879 with a 3-dimensional strong system coupled, via a single node, to a 3-dimensional weak system, Figure 4, and found negligible absolute differences in their voltage responses to complex spatio-temporal inputs while running 20 times faster than the original. We have achieved the strong-weak distinction through significant non-uniformity in the density of sodium channels. This was merely a matter of convenience. The effect can be achieved, see Golding et al. (2001) and Migliore and Shepherd (2002), by a large class of non-uniformities.

The critical assumption permitting our significant reduction is that the bulk of the neuron is weakly excitable - and this means that its response is well approximated by a quasi-active model. The delineation of such systems is of course wrapped up in the equally vexing questions of spike initiation and propagation. For neurons whose dendrites are not sufficiently weak to meet our definition we may apply our strong reduction to each branch and then invoke the activity measures of Rempe and Chopp (2006) and Rempe et al. (2008) to update these branches only when necessary. Regarding scope, our methods are equally suited to synaptic inputs modeled as conductance changes onto trees with arbitrary branching patterns and arbitrary non-uniform channel distributions, see Kellems et al. (2010) and Hedrick and Cox (2013), as well as to inputs via gap junctions, see Hedrick and Cox (2014). These methods can also be readily adapted to incorporate non-uniform distributions of spines and NMDA receptors as well as interaction with the cell's calcium handling machinery of channels, buffers, receptors and pumps.

### Conflict of interest statement

The authors declare that the research was conducted in the absence of any commercial or financial relationships that could be construed as a potential conflict of interest.
